# Аre Multivisceral Resections for Gastric Cancer Acceptable: Experience from a High Volume Center and Extended Literature Review?

**DOI:** 10.1055/s-0043-1761278

**Published:** 2023-02-03

**Authors:** Nikola Vladov, Tsvetan Trichkov, Vassil Mihaylov, Ivelin Takorov, Radoslav Kostadinov, Tsonka Lukanova

**Affiliations:** 1 Department of HPB Surgery and Transplantology, Military Medical Academy, Sofia, Bulgaria; 2 First Department of Abdominal Surgery, Military Medical Academy, Sofia, Bulgaria

**Keywords:** gastric adenocarcinoma, multivisceral resection, case series

## Abstract

**Introduction** Multivisceral resections (MVRs) in gastric cancer are potentially curable in selected patients in whom clear resection margins are possible. However, there are still uncertain data on their feasibility and safety considering short- and long-term results. The study compares survival, morbidity, mortality, and other secondary outcomes between standard and MVRs for gastric cancer.

**Materials and Methods** A monocentric retrospective study in patients with gastric adenocarcinoma, covering 2004 to 2020. Of the 336 operable cases, 101 patients underwent MVRs. The remaining 235 underwent standard gastric resections (SGRs), of which 173 patients were in stage T3/T4. To compare survival, a control group of 101 patients with palliative procedures was used—bypass anastomosis or exploration.

**Results** MVR had a lower survival rate than the SGR but significantly higher than the palliative procedures. The predominant gender in MVR was male (72.3%), with a mean age of 61 years. The perioperative mortality was 3.96% ( *n* = 4), and the overall median survival was 28.1 months. The most frequently resected organs were the spleen (67.3%), followed by the pancreas (32.7%) and the liver (20.8%). In 56.4% of the cases two organs were resected, in 28.7% three organs, and in 13.9% four organs. The main complication was bleeding (9.9%). The major postoperative complications in the MVR were 14.85%, and in the SGR 6.4% ( *p* < 0.05). Better long-term results were observed in patients who underwent R0 resections compared with R1.

**Conclusion** Multiorgan resections are characterized by poorer survival and a higher complication rate than gastrectomies. On the other hand, they have better long-term outcomes than palliative procedures. However, MVRs are admissible when performed by an experienced surgical team in high-volume centers.

Gastric cancer is the fifth most common cancer worldwide and the third most common cause of cancer-related death. 1 In Western populations, gastric cancers are often at an advanced stage at diagnosis, involving adjacent structures. 2 3 4 Patients with T4 tumors 5 often present with peritoneal dissemination or other distant metastases, while those in M0 stage are eligible surgical candidates. 6

The mainstay in managing locally advanced gastric carcinoma is radical surgery, with R0 multivisceral resection (MVR) being the only potentially curative treatment. 7 8 The Japanese Gastric Cancer Association 9 and National Comprehensive Cancer Network 10 recommend extensive resections of adjacent organs for T4b when negative margins are possible, while the European Society for Medical Oncology 11 advises neoadjuvant therapy. In addition, the achievement of R0 resection remains the most powerful predictor of long-term outcomes in patients who have undergone radical surgery for advanced gastric carcinoma. 12 However, the reliability and safety of the MVR in gastric carcinoma are still questionable because of insufficient and heterogeneous data available based on single-center retrospective studies. 13

## Material and Methods

The article presented a single-center retrospective study in patients with histologically confirmed gastric adenocarcinoma for the period between 2004 and 2020. This study represents nonconsecutive case series. Out of 366 resectable cases, MVR has been performed in 101 patients. The remaining 235 patients, 173 with tumor stage Т3 and Т4а, underwent standard gastric resection (SGR). The comparison between SGR and MVR was based on patients with stage Т3/Т4а carcinoma in the standard resection group. For survival, a comparison was included in a representative control group of 101 patients who underwent palliative procedures (PPs)—bypass anastomosis or explorative laparotomy. In none of the MVR cases was neoadjuvant chemotherapy performed. In the multivisceral group are not included splenectomies for iatrogenic lesions, extensive lymph node dissections, or technical issues. All operations in the MVR group were performed during the open approach, and subtotal resection or gastrectomy was achieved by the differentiation and distance of the tumor from the gastroesophageal junction according to the “Japanese gastric cancer treatment guidelines.” 9 Seventeen laparoscopic resections (7.2%) were achieved in the SGR group. MVR is performed when it is impossible to separate cancer from neighboring organs safely. T4b stage was provided preoperatively in 67% of cases. Most anastomoses were performed by standard suture technique in our department. There is a high volume in the performance of liver and pancreatic resections in our department, which greatly benefited us performing MVR. The postoperative period was closely monitored, and all complications were recorded. After discharge, the patients are followed up in the oncology clinic of our hospital at 6 months by computed tomography (CT) or positron emission tomography/CT and by examination of tumor markers (carcinoembryonic antigen, CA 19–9, and CA 72–4). The median follow-up period was 18 months (6–72). As we do not have sufficient data on the recurrence rate, we refer to survival under long-term outcomes. Obtained results were collected in a Microsoft Excel table. Statistical analysis was conducted using IBM SPSS StatisticsVer. 26 and MedCalc, Ver. 20.014. A *p* -value of < 0.05 was considered statistically significant. In the presence of normal distribution ( *p* > 0.05), metric variables were presented with mean value and standard deviation and analyzed using parametrical statistical methods. In the absence of normal distribution ( *p* < 0.05), we utilized median values and nonparametrical statistical methods. Correlation between qualitative variables was studied using chi-square analysis. This research is registered following the Declaration of Helsinki and attached to a publicly accessible database (ResearchRegistry; researchregistry7415; https://www.researchregistry.com/browse-the-registry#home/registrationdetails/61a6804bb59264001e08ffc9/ ). This case series has been reported in line with the PROCESS guideline. 14

## Results

**Fig. 1:**
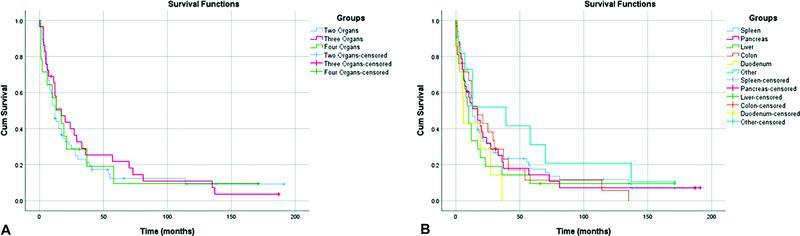
Comparing survival to the number ( **A** ) and type ( **B** ) of resected organs.

**Fig. 2:**
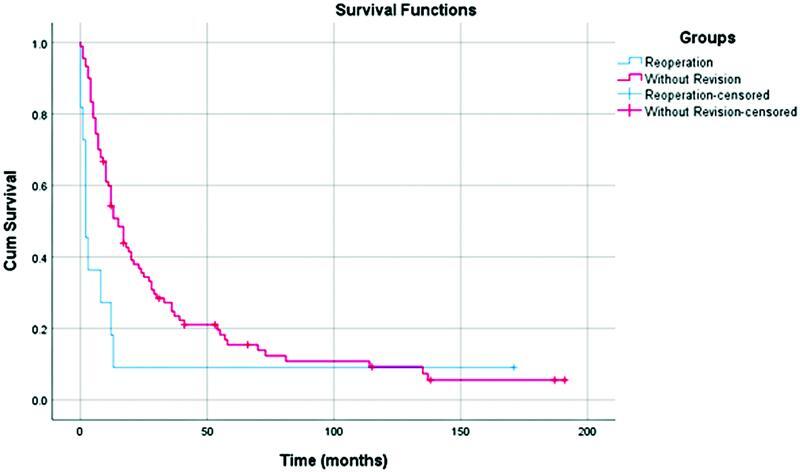
Comparison of survival according to reoperation in the multivisceral resection (MVR) group.

Most MVR patients were male (72.3%, *n* = 73), and the median patient age was 61 years ( Table 1 ). The median overall survival rate was 28.1 months. In 78.2% ( *n* = 79) of the patients, the main symptom was pain. The perioperative 30-day mortality rate was 3.96% ( *n* = 4). The complications in these four cases are bleeding in two patients, one esophageal-entero-anastomotic leakage, and one necrotizing pancreatitis with grade “C” postoperative pancreatic fistula. All are men with gastrectomy, cardiovascular diseases, and Eastern Cooperative Oncology Group performance status 1. In the first two patients, one adjacent organ was resected (spleen/liver), the next two (colon and liver), and in the last one, four organs (spleen, pancreas, colon, and liver). The perioperative mortality rate in SGR patients was 0.6% ( *n* = 1), significantly lower compared with that in MVR patients ( *p* = 0.046; 95% confidence interval [CI] –0.2123% to 9.1642%; chi-square test 3.975). The estimated annual survival rates were as follows: 1-year (58.3%), 3-year (27.7%), and 5-year (18.8%). In 5.9% ( *n* = 6) of patients, the survival was longer than 10 years. The median duration of postoperative hospital stay in MVR was 13 days compared with 10 days in SGR patients—another significant difference ( *p* < 0.0001; 95% CI 1.5715–4.4285). The most common resected organs during MVR were the spleen (67.3%, *n* = 68), pancreas (32.7%, *n* = 33), liver—wedge resection or left lateral lobectomy (20.8%, *n* = 21), colon (20.8%, *n* = 21), and duodenum/head of the pancreas—Whipple procedure (6.9%, *n* = 7). In 56.4% ( *n* = 57) of cases resection of 2 organs was performed (stomach + 1), in 28.7% ( *n* = 29) 3 organs, in 13.9% ( *n* = 14) 4 organs, and in 1 case (0.99%) were resected 5 organs (gastrectomy, distal spleno-pancreatectomy, left liver lobectomy, and left colectomy). There was no significant correlation between the number and type of resected organs and the rates of survival and major complications ( *p* > 0.05) ( Fig. 1A, B ). Borderline significance of survival rates was found only in cases with the Whipple procedure and in the subgroup “other organs” ( *p* = 0.05). The leading postoperative surgical complications in MVR were bleeding ( *n* = 10, 9.9%), intra-abdominal abscess ( *n* = 8, 7.9%), anastomotic insufficiency ( *n* = 6, 5.9%), and postoperative pancreatic fistula ( *n* = 6, 18.2% of 33 performed distal pancreatic resections) ( Table 2 ). Another significance was observed in the incidence of major postoperative complications (≥ IIIa) according to the Clavien–Dindo classification—14.85% ( *n* = 15) in MVR versus 6.4% ( *n* = 11) in SGR, respectively ( *p* < 0.0022; 95% CI 1.1236% to 17.1238%; chi-square test 5.269). In 21.8% ( *n* = 22) of patients the complications were treated conservatively, in 6.9% ( *n* = 7) by using minimally invasive techniques (endoscopic hemostasis or percutaneous drainage), while 10.9% ( *n* = 11) of cases required re-laparotomies (hemostasis – 8.9%, *n* = 9; re-anastomosis – 2.97%, *n* = 3). The comparison of survival following surgical revision in the MVR group revealed significantly lower survival rates than cases that did not require reoperation ( *p* = 0.028; 95% CI 0.6308% to 14.6966%; chi-square test 4.853) ( Fig. 2 ). No statistical correlation was found between postoperative bleeding and survival ( *p* = 0.18).

**Table 1:** Clinical characteristics and survival in MVR group

Parameters	*N* /% or Range
Gender	
Male	73 (72.3)
Female	28 (27.7)
Age (average)	61 (28–88)
Symptoms	
Pain	79 (78.2)
Weight loss	59 (58.4)
Weakness	45 (44.6)
Anemia	46 (45.5)
Vomiting	44 (43.6)
“En bloc” resection	74 (73.3)
Average postoperative stay (d)	13 (7–55)
Involved organs	
Spleen	68 (67.3)
Pancreas	33 (32.7)
Liver (wedge resection or left lobectomy)	21 (20.8)
Colon	21 (20.8)
Duodenum/Head of the pancreas (Whipple procedure)	7 (6.9)
Other (adrenal gland, ^1^ ovary, ^2^ esophagus, ^5^ small intestine, ^1^ diaphragm ^2^ )	11 (10.9)
Number of resected organs	
II	57 (56.4)
III	29 (28.7)
IV	14 (13.9)
V	1 (1)
Average survival (mo)	28.1 (3–135)
Annual survival	
1-y	58.3
3-y	27.7
5-y	18.8
30-d mortality	4 (3.96)

Abbreviation: MVR, multivisceral resection.

**Table 2:** Complications and treatment in MVR group

Parameters	*N* /%
Complications	
Bleeding	10 (9.9)
Abscess	8 (7.9)
Anastomotic leak	6 (5.9)
Pancreatic fistula	6 (5.9)
Postoperative pancreatitis	3 (2.97)
Dehiscence	4 (3.96)
Wound infection	5 (4.95)
Cardiopulmonary	12 (11.9)
Neurological	1 (0.99)
Sepsis	2 (1.98)
Clavien–Dindo classification	
II	15 (14.85)
IIIa	6 (5.94)
IIIb	5 (4.95)
IVa	3 (2.97)
IVb	1 (0.99)
≥ IIIa	15 (14.85)
Treatment	
Conservative	22 (21.8)
Mini-invasive	7 (6.9)
Reoperation	11 (10.9)
Hemostasis	9 (8.9)
Reanastomosis	3 (2.97)

Abbreviation: MVR, multivisceral resection.

The following analysis of pathologic characteristics in the MVR group showed that the average size of tumors, primarily located in the stomach body (37.6%, *n* = 38), was 66.8 mm ( Table 3 ). There was no association between tumor size and survival rates following the investigation of tumor diameter across 40 to 100 mm, at 10-mm intervals ( *p* > 0.05). The average number of lymph nodes dissected during surgery was 18, with slightly more than half of them (9.5) found to be metastatic. Poorly differentiated carcinomas were prevalent in the MVR group (60.4%, *n* = 61), typical considering tumor biology and invasiveness. The comparison of survival rates between patients with G3 versus G1/G2 carcinomas did not show significance ( *p* = 0.26). In 72.3% ( *n* = 73) of cases were positive for tumor invasion to another organ (Т4b), while the remaining 27.7% had no further organ involvement (Т3 or Т4а). The comparison of Kaplan–Meier estimates of overall survival between the two subgroups (T3/T4a vs. T4b) did not show a statistical difference ( *p* = 0.81). The prevalent lymph node status was N3 in 33.7% of cases, followed by N2 (30.7%) and N1 (16.8%). Only 18.8% of patients were without lymphatic invasion (N0). Clear resection margin (R0), the main target of surgical treatment, and a favorable prognostic factor were achieved in 84.2% ( *n* = 85) of cases.

**Table 3:** Pathological characteristics of multivisceral resections

Parameters	*N* /% or Range
Average lesion size (mm)	66.8 (25–145)
Primary tumor localization	
Cardia	18 (17.8)
Fundus	5 (5.0)
Corpus	38 (37.6)
Antrum	23 (22.8)
Linitis plastica	17 (16.8)
Degree of differentiation	
G1	4 (3.96)
G2	36 (35.6)
G3	61 (60.4)
T-stage	
Т3/T4a	28 (27.7)
Т4b	73 (72.3)
N-stage	
N0	19 (18.8)
N1	17 (16.8)
N2	31 (30.7)
N3	34 (33.7)
Harvested lymph nodes (average)	18 (5–61)
Metastatic lymph nodes (average)	9.5 (1–49)
Resection margin	
R0	85 (84.2)
R1	16 (15.8)

**Fig. 3:**
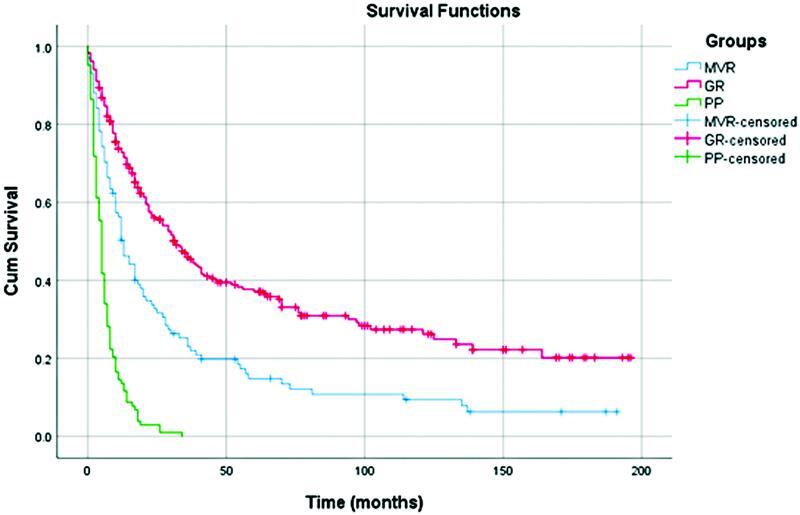
Comparison of survival between MVR, SGR, and PP. MVR, multivisceral resections; GR, gastric resections; PP, palliative procedures.

**Fig. 4:**
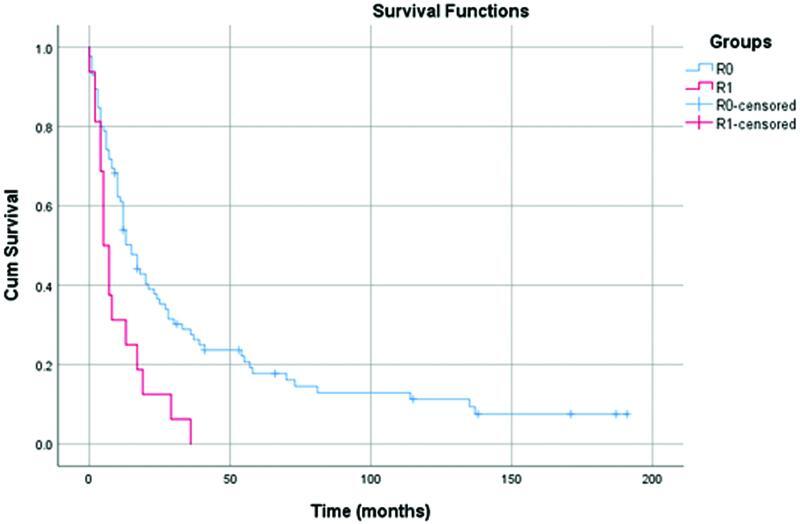
Kaplan–Meier in multivisceral resection (MVR) patients comparing survival according to R status.

Survival rates in the MVR group were lower than those in the SGR but still higher than in PP ( Fig. 3 ). The survival analysis showed no significant association with lymph node involvement (N– vs. N + , *p* = 0.2). Nevertheless, a comparison of lymph node status in 3-year survival has found a significantly lower survival rate in patients with N3 stage compared with N0 patients ( *p* < 0.022; 95% CI 4.8114% to 56.8151%; chi-square test 5.283). Long-term outcomes in MVR patients who underwent R0 resection were better than those with R1 ( *p* = 0.003; 95% CI 0.6678% to 17.1921%; chi-square test 4.635) ( Fig. 4 ).

**Fig. 5:**
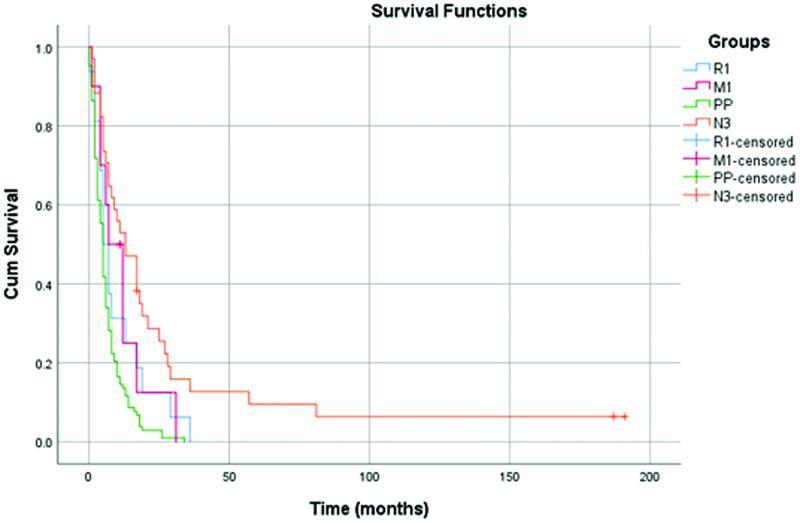
Comparison of survival between palliative procedure (PP), R1, M1, and N3 patients.

Comparing overall survival between MVR patients with R0 resection and SGR patients has shown a significance in favor of standard procedures ( *p* = 0.0002). On the other hand, another comparison between M1 disease (liver metastases), PP, and M0 stage has found that patients with PPs and metastatic disease are comparable in survival but statistically distinguishable from M0 cases. The comparison of survival rates in PPs, М1, R1, and N3 cases shows no significance ( *p* > 0.05) ( Fig. 5 ). Therefore, it can be concluded that R1 and N3 are independent prognostic factors for poor long-term outcomes.

## Discussion

Radical surgery can benefit patients with advanced gastric carcinoma, and R0 resection is significantly associated with better survival. 15 Nevertheless, long-term outcomes remain unsatisfactory. 16 Preoperative differentiation of Т3/T4a from Т4b lesions is performed by contrast-enhanced CT or endoscopic ultrasonography (EUS). However, neither of these methods has high specificity and sensitivity. 17 Study results show 80% estimated accuracy of CT for differentiation Т4 lesions, 18 while another analysis has demonstrated 79% overall accuracy of EUS. 19 Preoperative imaging diagnosis of adjacent organ invasion is not easy, and deciding on the performance of MVR is challenging even during surgery. 3 7 20 Adhesions caused by peritumoral desmoplastic reactions can be mistaken for carcinoma infiltration, especially in the pancreas. 3 21 Any adhesion suspicious of tumor invasion should be considered malignant during surgery, and adhesiolysis should be avoided. 22 Its performance is associated with a high risk for residual tumor tissue (R1) and unfavorable long-term outcomes. 6 Some Japanese case series have demonstrated that 55% of the tumors were considered infiltrative while being cancer mimicry, as confirmed by a pathological investigation. 16 23 Challenges in peri- and intraoperative detection of malignancies are the reason for the relatively low percentages of true tumor invasion (pT4b) reported in many series: Martin et al 13.8% 24 ; Xiao et al 39.7% 25 ; Molina et al 40% 26 ; Mita et al 46.3% 27 ; Yang et al 43.1% 22 ; Aversa et al 47.6% 28 ; Tran et al 53% 29 ; Dias et al 58.6% 13 ; Cheng et al 61.5% 30 ; Ozer et al 66.1%. 31 The true malignant invasion was detected in 72.3% ( *n* = 73) of cases in the present study, mainly attributed to delays in seeking medical care and late diagnosis of the disease (only 18.5%, *n* = 62 patients in the T1/T2 stage).

Many series demonstrate that the number and type of resected organs do not impact the short- and long-term outcomes. 22 32 33 34 35 The present study results confirm this finding ( *p* > 0.05) ( Fig. 1A, B ). Borderline significance was seen only in survival following Whipple procedures and the “other organs” subgroups ( *p* = 0.05). Pacelli et al report that the most common resected organ in MVR for gastric cancer was the spleen, while according to other authors, it is the pancreas. 13 26 In our study, the most common resected organ was the spleen (67.3%), followed by the pancreas (32.7%), liver (20.8%), and colon (20.8%). Splenectomies associated with iatrogenic injury or technical problems were excluded from the series. According to Tran et al, individual studies do not specify the number of splenectomies performed during MVR in connection with iatrogenic injury, technical issues, or extended lymph node dissection. 29 MVRs combined with liver or transverse colon resection are associated with better survival than MVR involving other adjacent organs. 30 36 37 In addition, DA Silva report that pancreatic involvement is associated with a poorer prognosis. 7 Our study does not confirm these findings. Some series suggest that splenectomy is an independent prognostic factor for poor survival, 38 39 while others have not made similar conclusions or reported heterogeneous findings depending on the disease stage. 16 24 34 40 When the removal of two or more organs is needed to achieve clear resection margins, the performance of MVR is justified despite coexisting perioperative risk. 6 Saito et al have demonstrated that in patients with achieved R0 resection, the number of resected organs does not impact the short- and long-term outcomes. 37

Several series report a higher incidence of surgical complications in patients who underwent MVR with two or more organs. 12 24 27 31 Some authors also report increased postoperative complications following MVR, 13 24 29 while others have not found statistical differences. 22 32 The reported rate of complications in MVR patients varies between 13.1 and 37.9%. 3 22 24 26 27 30 31 32 33 34 38 41 42 43 The number and type of resected organs in our series had no impact on the rate of complications. The comparison of major complications (Clavien–Dindo ≥ IIIa) in MVR and SGR patients has shown significance in the MVR group—14.85% versus 6.4% ( *p* < 0.002; 95% CI 1.1236% to 17.1238%; chi-square test 5.269). In a systematic review including 17 series and 734 patients who underwent MVR, Brar et al reported overall complication rates ranging from 11.8 to 90.5% and perioperative mortality from 0 to 15%. 6

Some authors emphasize the advantages of MVR based on evidence of an approximately 4% perioperative mortality rate, 24 26 30 32 44 while others report higher, significant rates in the range of 10 to 13%. 3 31 45 46 47 According to Mita et al, perioperative mortality varies between 1 and 15%. 27 In their studies, Pacelli et al, Yang et al, and Tran et al have not found statistical differences in perioperative mortality between patients who underwent MVR and SGR. 22 29 32 The results from our study showed a significance in postoperative mortality in the two groups, with a higher rate in the MVRs—3.96% versus 0.6% in standard resections ( *p* = 0.046; 95% CI –0.2123% to 9.1642%; chi-square test 3.975). Nevertheless, the observed mortality rate is relatively low compared with those reported in other series.

Dias et al have reported a significantly longer duration of hospital stay in the MVR group (an average of 17.7 vs. 11.5 days; *p* < 0.001), as well as a higher readmission rate. 13 We also observed a difference in the hospital stay in the MVR group (13 days) compared with the group with standard resections (10 days) ( *p* < 0.0001; 95% CI 1.5715–4.4285).

According to Kunisaki et al, a tumor diameter ≤ 100 mm and few metastatic lymph nodes (6 or less) are favorable indicators for curative MVR surgery for patients with gastric carcinoma. 42 On the other hand, Mita et al recommend radical MVR even in large tumors (> 100 mm) due to unsatisfactory outcomes from PPs. 27 Our analysis also confirmed the absence of correlation between short- and long-term outcomes and tumor size ( *p* > 0.05). The same refers to the lymph node status (N+ vs. N–; *p* > 0.05). Comparing lymph node status according to 3-year survival does not show any differences between N0 versus N1 ( *p* = 0.145; 95% CI –8.0677% to 53.4756%; chi-square test 2.128) or N0 versus N2 ( *p* = 0.159; 95% CI –7.4493% to 47.9259%; chi-square test 1.986). On the other hand, the comparison of N3 (≥ 7 lymph nodes) versus N0 revealed significantly lower survival rates in patients in the N3 stage ( *p* < 0.022; 95% CI 4.8114% to 56.8151%; chi-square test 5.283). This observation has been confirmed by other case series, 22 28 34 and some authors even concluded that MVR should not be performed in patients with extensive macroscopic lymph node involvement (N3). 22 23 34 42 46 The most common tumor invasion in MVR patients is lymphatic, vascular, and perineural. 13 Yang et al even suggest that tumor thrombosis is an independent prognostic factor since it could indicate micrometastases in the vessels and circulating tumor cells in the blood. 22 Further research in this area has shown that metastatic lymph nodes (lymph node ratio ≥ 2), peritoneal dissemination, distant metastases, and serum albumin levels (< 30 g/L) are independent poor prognostic factors. 41 46

The median overall survival reported in several series was between 13 and 38 months. 15 24 26 28 31 46 48 49 The median survival in our study was also in this range—28.1 months. Only one study demonstrated a statistically higher survival rate in MVR compared with SGR patients, 3 , while in the other series, the advantage favored SGRs. 13 15 24 50 Our analysis has shown statistically lower survival rates in the MVR group relative to the SGR group, but still significantly higher than those in the PP group ( *p* < 0.05) ( Fig. 3 ). Other studies have also confirmed the benefits of MVRs over PPs (explorative laparotomy or bypass anastomosis). 22 31 42 50 51 A negative resection margin (R0) is the most important and independent prognostic factor for favorable long-term outcomes. Several series demonstrate significant survival benefits from multivisceral R0 resections compared with R1-MVR. 3 , 17 , 21 , 22 , 24 , 26 , 28 , 30 , 31 , 37 Our analysis shows better long-term outcomes in patients who underwent R0 resection compared with R1 resection ( *p* = 0.003; 95% CI 0.6678% to 17.1921%; chi-square test 4.635) ( Fig. 4 ). Saito et al reported similar outcomes in patients with R1-MVR and patients with palliative interventions. 37 In contrast, Yang et al have demonstrated similar survival rates between patients with R1 resection and those who underwent PPs, and patients with N3 status. 22 The comparison between M1 (liver metastases), PP, and М0 cases revealed comparable outcomes in PP and M1 ( *p* < 0.05) but statistically different from those in М0 patients ( *p* > 0.05). Moreover, the summary comparison of overall survival in PP, М1, R1, and N3 cases shows no significance ( *p* > 0.05) ( Fig. 5 ). Therefore, we conclude that R1 and N3 are independent unfavorable prognostic factors for survival. Other multivariate analyses demonstrate that R and N status are the main prognostic factors in MVRs. 7 26

Cheng et al have reported better survival after MVR in patients with T3 compared with T4 tumors, 30 while other authors have not found statistically significant differences. 3 22 26 27 41 Real tumor invasion to another organ (Т4b) was established in 72.3% ( *n* = 73) of cases in our single-center series, while the remaining 27.7% were found in desmoplastic peritumoral mimicry. The comparison of Kaplan–Meier estimates of overall survival between the two subgroups (T3/T4a vs. T4b) did not show significance ( *p* = 0.81). A Chinese study from 2017 reports that Borrmann type IV gastric carcinoma is an independent prognostic factor for MVR. 52

Neoadjuvant chemotherapy can optimize patient selection for MVR. In addition, both adjuvant and neoadjuvant therapy in combination with MVR for gastric carcinoma could improve oncological outcomes. 7 Yang et al report that MVR followed by at least six chemotherapy courses extend median survival by nearly 22 months ( *p* < 0.05). 22 Neoadjuvant therapy is increasingly important in the treatment of gastric carcinoma. Unfortunately, in our series, there is only one patient with preoperative treatment. It is a consequence of no established standard for neoadjuvant therapy for gastric carcinoma in our country, which necessitated the performance of initial surgery with subsequent adjuvant treatment. That is a significant shortcoming that we have nearly managed to eliminate. Neoadjuvant treatment should be an integral part of locally advanced gastric tumors, leading to better long-term outcomes such as overall and disease-free survival.

The retrospective nature of the study can be pointed out as a disadvantage. In addition, the quality of life and disease-free period of multiple patients were not monitored due to low collaboration. Another issue is that in a certain percentage of cases, pathologists have not determined the status of the resection lines. Аlso did not specify the exact lymph status due to the specimen's poor examination of all available lymph nodes. We are currently trying to unravel these issues and hope that in the future, we will be able to deal with them to improve the accuracy of our results. In addition, we allow for the possibility of statistical error type II as a limitation of the study.

## Conclusion

MVRs are associated with poorer survival and a higher incidence of complications than SGRs. On the other hand, they are associated with better long-term outcomes than palliative interventions. Achievement of negative resection margins is a main independent factor for better survival, while R1 and N3 status and reoperations are factors for unfavorable long-term outcomes. However, the limited number of studies and their heterogeneous results do not permit any general conclusion about the safety of multiorgan procedures. Nevertheless, it can be concluded that MVRs are admissible when performed by experienced surgical teams at high-volume centers.
